# High confidence proteomic analysis of yeast LDs identifies additional droplet proteins and reveals connections to dolichol synthesis and sterol acetylation[S]

**DOI:** 10.1194/jlr.M050229

**Published:** 2014-07

**Authors:** Erin Currie, Xiuling Guo, Romain Christiano, Chandramohan Chitraju, Nora Kory, Kenneth Harrison, Joel Haas, Tobias C. Walther, Robert V. Farese

**Affiliations:** * Department of Biochemistry and Biophysics University of California, San Francisco, CA 94158; ** Department of Medicine, University of California, San Francisco, CA 94158; † Gladstone Institute of Cardiovascular Disease, San Francisco, CA 94158; § Department of Cell Biology, Yale University, New Haven, CT 06520

**Keywords:** lipid droplets, lipid metabolism, lipids, protein targeting, polyprenol synthesis

## Abstract

Accurate protein inventories are essential for understanding an organelle’s functions. The lipid droplet (LD) is a ubiquitous intracellular organelle with major functions in lipid storage and metabolism. LDs differ from other organelles because they are bounded by a surface monolayer, presenting unique features for protein targeting to LDs. Many proteins of varied functions have been found in purified LD fractions by proteomics. While these studies have become increasingly sensitive, it is often unclear which of the identified proteins are specific to LDs. Here we used protein correlation profiling to identify 35 proteins that specifically enrich with LD fractions of *Saccharomyces cerevisiae*. Of these candidates, 30 fluorophore-tagged proteins localize to LDs by microscopy, including six proteins, several with human orthologs linked to diseases, which we newly identify as LD proteins (Cab5, Rer2, Say1, Tsc10, YKL047W, and YPR147C). Two of these proteins, Say1, a sterol deacetylase, and Rer2, a *cis*-isoprenyl transferase, are enzymes involved in sterol and polyprenol metabolism, respectively, and we show their activities are present in LD fractions. Our results provide a highly specific list of yeast LD proteins and reveal that the vast majority of these proteins are involved in lipid metabolism.

The lipid droplet (LD) is a cytoplasmic organelle that is ubiquitous among eukaryotic cells and is also found in some prokaryotic cells (1–3). LDs were long thought to be mostly inert, but are now recognized as a bona fide organelle with dynamic size, number, distribution, and protein composition (4). LD proteins include a family of structural proteins (5), many enzymes involved in lipid metabolism, and an assortment of proteins with other functions. Protein localization to the LD can be regulated by many factors [e.g., development regulates histone localization (6) and phospholipid content regulates CCT1 localization (7)]. A thorough understanding of protein composition is an essential step in understanding the functions of the LD.

The LD has a unique architecture of a neutral lipid core bounded by a phospholipid monolayer. The surfactant monolayer imposes specific structural requirements on proteins localized to the LD, i.e., prohibiting transmembrane proteins with luminal domains and favoring structures that can localize to the interface by dipping segments into the hydrophobic phase, such as proteins with hydrophobic sequences (8, 9) or amphipathic helices (5). LD biogenesis and growth are uniquely dependent on neutral lipid synthesis because the organelle core contains primarily TGs and sterol esters (SEs), with composition varying by cell type and nutritional status. In yeast, the composition of TG and SE is roughly 50% for each (10).

LDs are often found in close apposition to other organelles, including peroxisomes (11), mitochondria (12–14), endosomes (15), phagosomes (16), and especially the endoplasmic reticulum (ER) (17, 18), which is likely their site of origin [reviewed in (19, 20)]. In fact, some proteins that appear to target the LD may actually target ER membranes closely apposed to the LD; this can be difficult to distinguish at the resolution of confocal light microscopy (∼300 nm). The close association of LDs with other organelles makes their biochemical purification challenging. Additionally, the hydrophobic nature of the organelle offers a potential sink for non-LD proteins whose topologies are disrupted during the mechanical fractionation process. These artifacts, combined with the high sensitivity of MS, often yield LD proteomes with low specificity.

We sought to determine a high-confidence proteome of the yeast *Saccharomyces cerevisiae* LD, an established model for LD studies (21). Although comprehensive lists of yeast LD proteomes have been reported (11, 22), there is little overlap between these lists and it remains unclear which of the candidate proteins identified by proteomics are specific to LDs. We sought to overcome the specificity limitations of LD proteomes by using protein correlation profiling (PCP), a quantitative method of determining purification profiles of proteins compared with organelle markers, based on high-resolution MS. PCP was successfully used to create specific inventories of many organelles (23, 24), including LDs in *Drosophila melanogaster* cells (25). We reasoned that bona fide LD proteins should fulfill two criteria: *a*) enrichment in the LD purification fraction by PCP, and *b*) localization to LDs by microscopy. We used PCP to generate a high-confidence list of 35 proteins that specifically copurify with the yeast LD. By cross-referencing with fluorescence microscopy in this study or previous reports, we verified that 30 of these proteins are bona fide LD proteins. We showed that two proteins (Faa1 and Hfd1), previously identified in yeast LD proteomes, in fact, do localize to LDs. Additionally, we identified six new LD proteins (Cab5, Rer2, Say1, Tsc10, YKL047W, and YPR147C), and we assessed enzymatic activities for two of these proteins, Say1 and Rer2, at LDs.

## EXPERIMENTAL PROCEDURES

### Strains, media, and materials

*S. cerevisiae* strain BY4741 (*MAT****a*** *his3*Δ*0 leu2*Δ*0 met15*Δ*0 ura3*Δ*0)* was used as WT. Yeast strains were routinely transformed using lithium acetate. Cells were cultured in synthetic complete media with dextrose containing 2% dextrose, 0.67% yeast nitrogen base (BD Biosciences), amino acids (Sunrise Science), and ammonium sulfate. Cells were grown for 2 days at 30°C to stationary growth phase for all experiments.

### Protein localization

C-terminally tagged green fluorescent protein (GFP) strains were created using published cloning cassettes (26). PCR primers were designed using Primer3 software and purchased from Elim Biopharm. Yeast were labeled with 1:1,000 monodansyl pentane (MDH) (Abgent) for LD identification (27) and allowed to settle on concanavalin A-coated coverslips for 10 min. They were then mounted on slides and images were acquired by using a Nikon ECLIPSE Ti 2000 microscope with Yokogawa CSU-X1 spinning disk and Hamamatsu ImagEM electron multiplier CCD camera with image acquisition and mechanical control by Micro-Manager. Solid-state lasers at excitation/emission of 405/460 nm, 491/520 nm, and 561/595 nm were used. Images were deconvolved (Huygens SVI) and cropped (Image J). The fraction of LDs with GFP was determined manually by counting numbers of LDs with and without GFP signal colocalization. The fraction of GFP colocalizing with LDs was determined by a custom CellProfiler pipeline and Python script that calculated the fraction of total GFP intensity that colocalized with MDH punctae on a per cell basis.

Localization was confirmed by crude cellular fractionation and immunoblotting, using mouse monoclonal anti-protein disulfide isomerase (Abcam), anti-GFP (Roche), and rabbit polyclonal anti-hemaggutinin (HA) (Upstate). For crude fractionation (e.g., in experiments of Fig. 5A, C and ), cells were dounce-homogenized and lysates spun at 300 *g* for 30 min and then 100,000 *g* for 30 min. LDs were collected with a tube slicer (Beckman Coulter) and the remaining supernatant and pellet were collected.

### TLC

Lipids were extracted by bead beating cells in water:CHCl_3_:methanol (0.3:1:1), collecting the single phase, and drying it under N_2_ gas. Lipids were resuspended in chloroform, separated on silica gel TLC plates (Whatman or Analtech) using hexane:ethyl ether:acetic acid (80:20:1), and detected by charring with cuprous sulfate. Bands were identified by comparison to standards.

### LD purification

Stable isotope labeling by amino acids in cell culture (SILAC) was performed (28, 29). Cells were pelleted, washed with water, and then incubated in 0.1M Tris-Cl (pH 9) and 10 mM DTT at 30°C for 10 min. They were washed and resuspended in 20 mM KH_2_PO_4_ (pH 7.4) and 1.2M sorbitol to 0.5 g/ml, and digested with 4 mg/g zymolyase 100T (MP Biomedicals) at 30°C for 2 h. Cells were pelleted, washed, and resuspended in 5 ml 20 mM HEPES (pH 7.4), 0.6M sorbitol, 1 mM EDTA, and EDTA-free protease inhibitor pellet (Roche) and homogenized in a dounce homogenizer for 40 strokes. Homogenized cells were spun at 300 *g* for 30 min, 20,000 *g* for 30 min, and then 100,000 *g* for 30 min with the pellet collected at every centrifugation step. The supernatant was then floated through a sucrose gradient by centrifuging overnight at maximum speed in an SW41 rotor. The LDs were collected with a tube slicer (Beckman Coulter) and other fractions were collected by pipette. Six gradient fractions and the three pellets from the initial high-speed spins were analyzed by MS.

### LC-MS/MS analysis

Each peptide fraction was separated by reversed-phase chromatography on a Thermo Easy nLC 1000 system connected to a Q Exactive mass spectrometer (Thermo) through a nano-electrospray ion source. Peptides were separated on 15 cm columns (New Objective) with an inner diameter of 75 μm packed in-house with 1.9 μm C18 resin (Dr. Maisch GmbH). Chromatographic runs (120 min) were used and peptides were eluted with a linear gradient of acetonitrile from 5 to 30% in 0.1% formic acid for 95 min at a constant flow rate of 250 nl/min. The column temperature was kept at 35°C. Eluted peptides were directly electrosprayed into the mass spectrometer. Mass spectra were acquired on the Q Exactive in a data-dependent mode to automatically switch between full scan MS and up to 10 data-dependent MS/MS scans. The maximum injection time for full scans was 20 ms with a target value of 3,000,000 at a resolution of 70,000 at *m/z* 200. The 10 most intense multiple charged ions (z ≥ 2) from the survey scan were selected with an isolation width of 3 thomson and fragmented with higher energy collision dissociation with normalized collision energies of 25. Target values for MS/MS were set to 1,000,000 with a maximum injection time of 120 ms at a resolution of 17,500 at *m/z* 200. To avoid repetitive sequencing, the dynamic exclusion of sequenced peptides was set to 20 s.

The resulting MS and MS/MS spectra were analyzed using MaxQuant (version1.3.0.2), utilizing its integrated ANDROMEDA search algorithms (30, 31). Peak lists were searched against local databases for *S.* *cerevisiae* (obtained from the *Saccharomyces* Genome Database, Stanford University; 6,641 entries, July 26, 2012) with common contaminants added. The search included carbamidomethlyation of cysteine as fixed modification, and methionine oxidation and N-terminal acetylation as variable modifications. Maximum allowed mass deviation for MS peaks was set to 6 parts per million, and 20 parts per million for MS/MS peaks. Maximum missed cleavages were two. The false discovery rate was determined by searching a reverse database. Maximum false discovery rates were 0.01 both on peptide and protein levels. Minimum required peptide length was six residues. Proteins with at least two peptides (one of them unique) were considered identified. The “match between runs” option was enabled with a time window of 2 min to match identification between replicates. Soft-clustering was performed using the Mfuzz software package in the statistics software program R. The cluster number C was set at 9, and cluster stability m = 1.6.

### Criteria for defining LD proteins

**Fig. 1.**
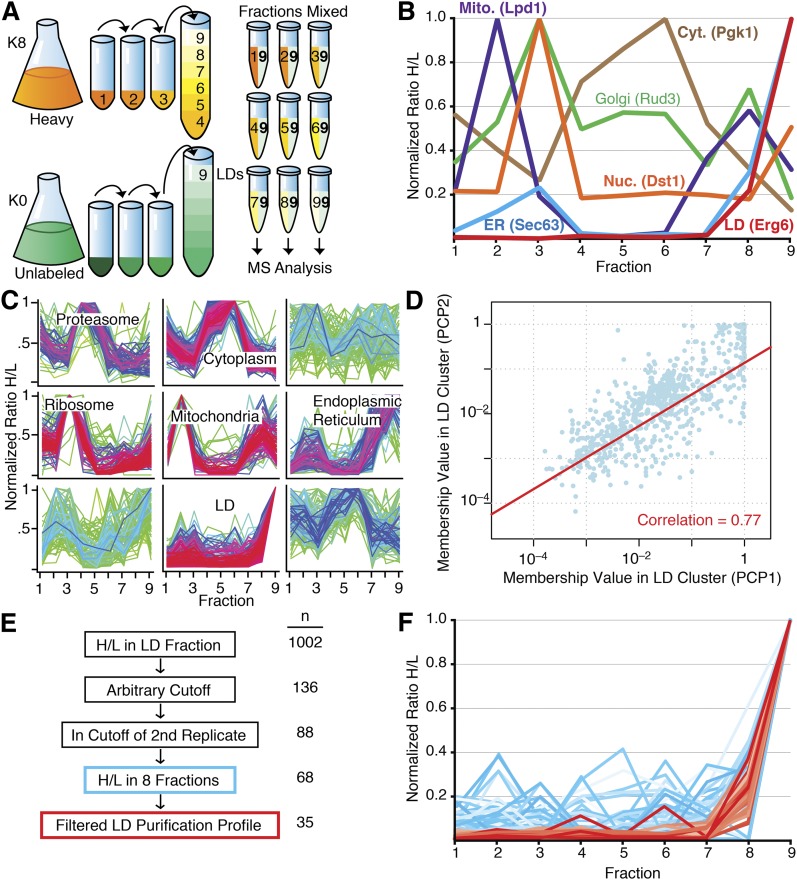
Identification of LD proteins in *S. cerevisiae* using PCP. A: Schematic of PCP workflow. B: Purification profiles of representative proteins for different organelles. Mito., mitochondria; Cyt., cytoplasm; Nuc., nucleus. C: Soft clustering of all fractions of a LD purification. Proteins identified in the LD fraction that were identified in at least five of the nine fractions were analyzed. A normalized H/L was used. Clusters that showed enrichment of proteins of a certain organelle or function are indicated. Proteins with a minimal membership value of 0.1 for LD cluster are shown in supplementary Table II. D: Reproducibility of LD proteome data between experiments. Pearson correlation = 0.77. E: Schematic of data filtration to create high-confidence LD protein list. F: Proteins identified with high confidence in the LD PCP cluster of two biological replicates. Proteins in red passed additional stringent filtration criteria whereas those in blue did not.

To define a high-confidence LD protein set, we applied four criteria to filter candidates. First, we set an arbitrary cutoff point for rank-ordered membership in the LD cluster as the top 136 proteins (see supplementary Table II for a list of all proteins with a LD cluster value >0.1). We chose this arbitrary point because it included the majority of previously identified LD proteins in two experimental replicates. Second, proteins had to be above the arbitrary cutoff in both experimental replicates. Third, proteins had to have a calculable heavy/light ratio (H/L) in at least eight fractions so that we were confident that their cluster membership value was not strongly influenced by missing data points. Finally, proteins were filtered based on a purification profile that closely matched that of 12 well-established LD proteins (Fig. 3A). In the case of our analysis, these stringent criteria used the thresholds of H/L <0.06 in fractions 1–3, <0.16 in fractions 4–6, <0.2 in fraction 7, and <0.371 in fraction 8 (Fig. 1E). By including these selected thresholds, we were able to maximize specificity of the selected LD proteins.

### *cis*-Isoprenyl transferase assay

Cells were crudely fractionated as above. To reduce cytoplasm in the LD fraction, LDs in 1.5 ml Eppendorf tubes were rinsed twice by addition of 1 vol buffer, spun at maximum speed in a tabletop centrifuge, and a needle and syringe were inserted below the floating LDs to remove 1 vol cytoplasm. Pellets were rinsed twice and then resuspended in yeast *cis*-isoprenyl transferase (*cis*-IPTase) reaction buffer [60 mM HEPES (pH 8.5), 5 mM MgCl_2_, 2 mM DTT, 2 mM NaF, and 2 mM sodium orthovanadate]. Yeast reaction mixtures contained 100 μg protein, 50 μM farnesyl pyrophosphate (FPP), and 45 μM ^14^C-isopentyl pyrophosphate (IPP) (American Radiolabeled Chemicals). Reactions were incubated at 30°C for 1 h and quenched by addition of 2 ml CHCl_3_:methanol (2:1). Products were separated from unreacted water-soluble IPP by partition through addition of 0.8 ml of 0.9% NaCl in water. The organic phase was washed three times with CHCl_3_:methanol:water (3:48:47) and dried under nitrogen. The dried sample was resuspended in hexane and loaded onto silica gel TLC plates, run in hexane:ethyl acetate (80:20), and dried. Plates were exposed to a phosphor screen for 3–4 days and compared with iodine-labeled standards. The band corresponding to dolichol was scraped from the TLC plate, resuspended in scintillation counting liquid, and counted in a scintillation counter.

### Cholesteryl acetate deacetylase assay

Cells were crudely fractionated, as described above, in lysis buffer of 200 mM sorbitol, 10 mM HEPES-KOH (pH 7.5), 100 mM NaCl, 5 mM MgCl_2_, and 1 mM EDTA. Microsome (300 ug) or cytoplasm (300 μg) and LD protein were assayed for cholesteryl acetate (CA) deacetylase activity as described in (32). Cell fractions were incubated with 0.143 μCi ^14^C-CA (American Radiolabeled Chemicals) in 26 nmol total CA for 1 h at 30°C. The assay was stopped by adding 1.5 vol of CHCl_3_. Three volumes of methanol were added and the reaction was vortexed until clear. Ortho-phosphoric acid (2.8%, 1.5 vol) was added and the samples were centrifuged for 2 min at 13,000 rpm. The upper aqueous layer was discarded. Samples were re-extracted with 1.5 vol CHCl_3_ and 3 vol acidified water. The lower organic layer was collected and dried under nitrogen. The dried sample was resuspended in CHCl_3_:methanol (1:1), loaded onto silica gel TLC plates, run in petroleum ether:diethyl ether:acetic acid (70:30:2), and dried. Plates were exposed to a phosphor screen for 2–3 days and compared with iodine-labeled standards.

## RESULTS

### PCP yields a distinct LD purification profile

LDs were isolated from WT yeast grown at 30°C to stationary phase, where LDs are most abundant. To obtain the fractions for PCP, we purified LDs from cells labeled with heavy nonradioactive isotope-containing lysine [stable isotope labeling by amino acids in cell culture, SILAC (28)] using sequential differential centrifugation and a sucrose density gradient. We collected samples from pellets of each initial centrifugation (fractions 1–3) and six layers of the sucrose density gradient (fractions 4–9). By using peptides identified in the various fractions, we found that the sequential centrifugations pelleted primarily the following: *a*) unbroken cells and agglomerated membrane in fraction 1; *b*) vacuolar, nuclear, ER, plasma, endosomal, and Golgi membranes in fraction 2; and *c*) transport vesicles, endosomal membranes, and Golgi complex in fraction 3. The sucrose density gradient separated the cytoplasm into six additional fractions. We combined each of these samples with equal amounts of protein from the LD fraction (fraction 9) of unlabeled cells and analyzed the combined samples by liquid chromatography coupled online to electrospray ionization and high-resolution MS/MS (**Fig. 1A**). We measured 16,589 peptides from 2,165 proteins in all fractions with 1,377 proteins identified in the LD fraction, 993 of which had calculable H/Ls (supplementary Table I).

We calculated H/Ls for each protein in every fraction. To compare individual proteins, we set the maximum H/L to one and normalized the ratios in other fractions to this H/L. To directly compare different organelle purification profiles, we plotted a representative profile for a protein from each of the major cellular organelles, choosing proteins that were only annotated to a single intracellular compartment in the *Saccharomyces* Genome Database (Fig. 1B). We found that bona fide LD proteins peak in fraction 9 with very little representation in other fractions. ER proteins, which are especially difficult to separate from LDs, similarly peak in fraction 9, but also have smaller peaks in fractions 2 and 3. Several other organelles have abundant proteins in the LD fraction, highlighting the need for a technique such as PCP to determine specificity.

We used soft clustering to generate clusters of typical purification profiles with each protein assigned a membership value for each cluster (33). The membership value provides a measurement of the similarity between each protein’s purification profile and the cluster, thereby providing a measure of the likelihood that a protein belongs to a certain cluster. Soft clustering identified a group of LD proteins (Fig. 1C, bottom center) as well as clusters containing mostly proteins from other organelles [e.g., mitochondria (center) and ER (center right)] (see supplementary Table II for all proteins in the LD cluster with H/L >0.1).

To assess reproducibility of the LD PCP measurements, we repeated the purification and analysis and compared the membership value for the LD cluster of each identified protein. The results were highly reproducible, with a Pearson’s correlation of 0.77 between the two PCP experiments (Fig. 1D). We performed the same comparison with a third biological replicate and found that it correlated well with both previous PCPs (*r* = 0.79 and *r* = 0.76, respectively), again showing the reproducibility of our technique and dataset. Thus, with respect to the high-confidence list, the results were highly reproducible.

We applied an arbitrary cutoff to the LD cluster (as described in Experimental Procedures) and filtered the list to include only proteins that were within this arbitrary cutoff in a biological replicate to generate a cluster of 88 proteins that reproducibly purify in the LD fraction (filtration schematic in Fig. 1E). To further separate bona fide LD proteins, we applied stringent filtration criteria to the cluster to separate it into highest-confidence LD proteins (Fig. 1F, red) and suspected non-LD proteins (Fig. 1F, blue). This analysis yielded a list of 35 highest-confidence LD proteins (**Table 1**).

**TABLE 1.** Identification of 35 proteins that specifically purify with the LD

ORF	Gene	Function	Proteomes Present	Microscopically Localized to LD	Biochemically Localized to LD
YIL124W	AYR1	Acyl-DHAP reductase, catalyzes lyso-PA formation	A, B, G	(69)	(60)
**YDR196C**	**CAB5**	**Dephospho-CoA kinase involved in CoA synthesis**	—	**Current Work**	—
YOR245C	DGA1	Diacylglycerol acyltransferase, catalyzes DAG to TAG	G	(69)	(63)
*YHL030W*	*ECM29*	*Proteasome assembly*	—	—	—
YBR177C	EHT1	Acyl-CoA:ethanol acyltransferase	A, B, G	(69)	—
YGR175C	ERG1	Squalene epoxidase, enzyme in ergosterol synthesis	A, B, G	(68)	(68)
YLR100W	ERG27	3-keto sterol reductase, enzyme in ergosterol synthesis	B,G	(69)	(66)
YML008C	ERG6	24-C-sterol methyltransferase, enzyme in ergosterol synthesis	A, B, G	(68)	(64)
YHR072W	ERG7	Lanosterol synthase, enzyme in ergosterol synthesis	A, B, G	(65)	(65)
YOR317W	FAA1	Fatty acyl-CoA synthetase, activates imported FAs	A, B, G	Current Work	—
YMR246W	FAA4	Fatty acyl-CoA synthetase, activates imported FAs	A, B, G	(69)	—
YBR041W	FAT1	Fatty acyl-CoA synthetase, activates imported FAs	A, B, G	(69)	—
*YIR038C*	*GTT1*	*Glutathione transferase*	B, G	—	(22)
YMR110C	HFD1	Fatty aldehyde dehydrogenase	B, G	Current work	—
YBR204C	LDH1	Serine hydrolase, weak TG lipase activity	G	(70)	(70)
*YML059C*	*NTE1*	*Phospholipase B*	—	—	—
YDL193W	NUS1	Putative prenyltransferase involved in dolichol synthesis	A, G	(43)	—
YKR046C	PET10	Unknown	A, B, G	(69)	—
YPL206C	PGC1	Phosphatidyl glycerol phospholipase C, catalyzes PG to DAG	G	(71)	(72)
*YGR233C*	*PHO81*	*Cyclin-dependent kinase inhibitor*	—	—	—
**YBR002C**	**RER2**	**Prenyltransferase involved in dolichol synthesis**	—	**Current work**	**Current work**
**YGR263C**	**SAY1**	**Sterol deacetylase**	—	**Current work**	**Current work**
YDL052C	SLC1	Acyltransferase, catalyzes lyso-PA to PA	A, B, G	(69)	(61)
*YPR140W*	*TAZ1*	*Lyso-PC acyltranferase, catalyzes lyso-PC to PC*	—	—	—
YKL140W	TGL1	Lipase, catalyzes SE to sterol	A, B, G	(50)	(73)
YMR313C	TGL3	Lipase, catalyzes TAG to DAG, DAG to MAG, and LPA to PA	A, B, G	(74)	(74)
YKR089C	TGL4	Lipase, catalyzes TAG to DAG, LPA to PA, and SE to sterol	G	(75)	—
YOR081C	TGL5	Lipase, catalyzes TAG to DAG and LPA to PA	G	(75)	(75)
**YBR265W**	**TSC10**	**3-Ketosphinganine reductase, involved in sphingosine synthesis**	**G**	**Current work**	—
YML013W	UBX2	Involved in ER-associated protein degradation, regulates LD homeostasis.	G	(76)	(22)
YMR152W	YIM1	Unknown	A, G	(69)	—
YKL094W	YJU3	Monoacylglyceride lipase, catalyzes MAG to glycerol	A, G	(69)	(67)
**YKL047W**	—	**Unknown, putative lipase**	—	**Current work**	—
YOR059C	—	Unknown, putative lipase.	A	(69)	—
**YPR147C**	—	**Unknown**	—	**Current work**	—

Identified proteins were found as described in the Results and are annotated for ORF, gene name, presence in other proteomes, and previous localization to the LD by microscopy or biochemistry. Proteomes are abbreviated as A = Athendstaedt et al., 1999 (37), B = Binns et al., 2006 (11), and G = Grillitsch et al., 2011 (22). Proteins were considered previously identified if they were previously microscopically or biochemically localized to the LD or if they were previously identified in more than one proteome. Newly identified proteins are bolded in the table. Proteins that have not been verified by microscopy to localize to the LD in our study or others are in italics. DAG, diacylglycerol; DHAP, dihydroxyacetone phosphate; LPA, lysophosphatidic acid; MAG, monoacyl glycerol; PA, phosphatidic acid; PC, phosphatidyl choline; PG, phosphatidyl glycerol; TAG, triacylglycerol.

### Verification of LD protein localization

**Fig. 2.**
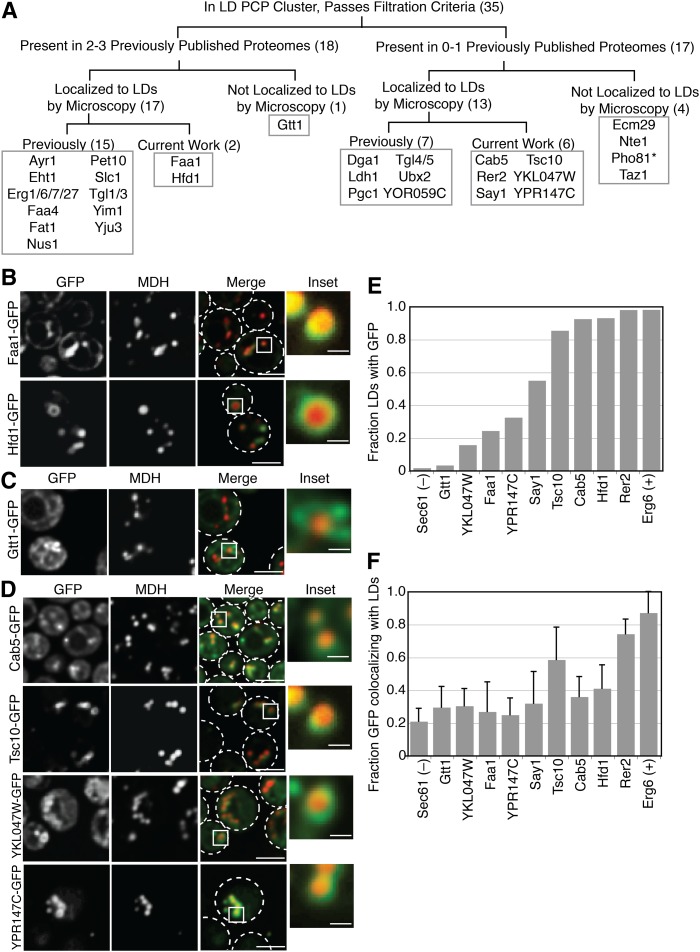
Identification and verification of LD proteins. A: Logic flow diagram for identification of LD proteins. Numbers in parentheses refer to the number of proteins in each category. *Indicates protein that was not imaged. B: Verification of LD localization of two proteins previously found in multiple proteomes, but not previously localized to the LD by microscopy. C: Microscopy of a protein that reproducibly purifies with LDs but does not appear to localize to the LD. D: Microscopy of four newly identified LD proteins. E: Quantification of the fraction of LDs that colocalize with GFP on a population basis. Sec61 and Erg6 are negative and positive controls, respectively. Greater than 150 LDs/genotype were quantified. F: Quantification of the fraction of GFP signal that colocalizes with LDs on a per cell basis. Sec61 and Erg6 are negative and positive controls, respectively. Greater than 50 cells/genotype were quantified. Error bars are standard deviation. GFP tagged proteins were colocalized with MDH, a LD marker vital dye. Cells were grown in synthetic complete media with dextrose to stationary phase. The scale bar is 3.5 μm on merged images and 0.7 μm on inset images.

We next tested to determine which proteins in the high-confidence PCP list are bona fide LD proteins, as confirmed by localization to LDs by spinning disk live-cell fluorescence microscopy (**Fig. 2B–D**, quantified in Fig. 2E, F). We consider a protein a bona fide LD protein if it is present at the LD, regardless of where else within the cell it is also present. Of the 35 identified proteins, 19 were previously identified as LD proteins by fluorescence microscopy in published works (annotated in Table 1 and Fig. 2A). Three proteins (Faa1, Hdf1, and Gtt1) were identified in multiple other yeast LD proteomes, but their localization to the LD by fluorescence microscopy was not explored. To assess their localization, we tagged these proteins at the C terminus with GFP to assess LD localization in stationary growth phase by colocalization with MDH, a blue LD vital dye. Hfd1-GFP exhibited predominantly LD localization while Faa1-GFP appeared to be visible in additional cellular compartments, likely the ER (representative images shown in Fig. 2B, quantified in Fig. 2E, F). Gtt1-GFP was predominantly present in other cellular compartments (Fig. 2C), however quantification of the microscopy revealed that very few LDs had GFP signal (Fig. 2E), but the fraction of GFP signal that colocalized with LDs was comparable to other bona fide LD proteins (Fig. 2F), which could explain why Gtt1 biochemically fractionates with LDs.

Seventeen proteins identified in our PCP proteome were not previously annotated to the LD or, in some cases, were only found in a single report of the LD proteome. Seven of these proteins were previously localized to the LD by microscopy (Dga1, Ldh1, Pgc1, Tgl4, Tgl5, Ubx2, and YOR059C). Of the 10 remaining proteins, three (Ecm29, Nte1, and Taz1) did not show LD localization when we examined C-terminal GFP fusions of them by microscopy, suggesting that they purify with the LD fraction, but do not localize to this organelle. We were unable to image Pho81 because we could not express the tagged protein. However, we newly identified six proteins (Cab5, Rer2, Say1, Tsc10, YKL047W, and YPR147C) as localizing to LDs (Fig. 2D–F).

### Comparison of PCP proteome to reported yeast LD proteomes

**Fig. 3.**
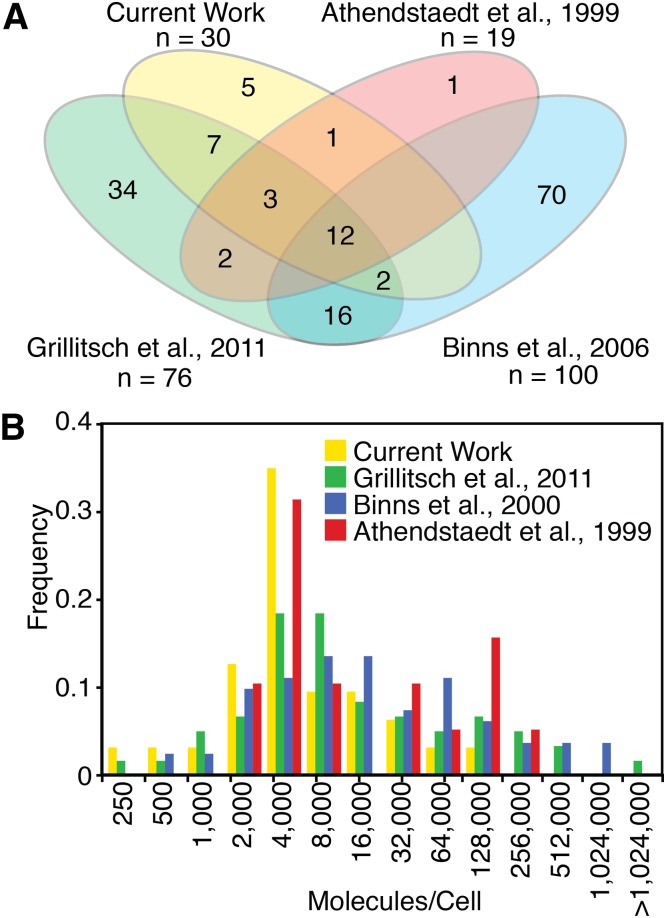
Comparison of current LD proteome with previously reported yeast LD proteomes. A: Venn diagram showing overlap of LD-annotated proteins between the current work and three previously reported yeast LD proteomes that attempted specificity. B: Current work using PCP for identification of LD proteins has fewer high-abundance proteins than previously reported yeast LD proteomes. Protein abundances are from Ghaemmaghanmi et al. 2003 (77).

Of the 959 proteins with a calculable H/L in the LD fraction, we found 35 (<4%) that specifically purify with the LD according to our filtering criteria (see Experimental Procedures) (Table 1). We compared our LD protein list to other reported proteomes that attempt to threshold their lists and found that the current proteome overlaps most (95%) with the one reported by Athendstaedt et al. (34), which utilized earlier and less sensitive proteomics technology. In contrast, there was considerably less overlap with LD proteomes generated more recently with more sensitive MS techniques (Grillitsch et al. (22), 55%; Binns et al. (11), 30%) (**Fig. 3A**).

Twelve proteins (Ayr1, Eht1, Erg1, Erg6, Erg7, Faa1, Faa4, Fat1, Pet10, Slc1, Tgl1, and Tgl3) were detected in the current work and all reported proteomes. Eleven of them are known to be lipid metabolic enzymes.

We also analyzed our results in relationship to protein abundance within cells, using data from Ghaemmaghanmi et al. (35) (Fig. 3B). Previously reported yeast LD proteomes show an overrepresentation of high abundance proteins, suggesting more contamination of the LD fraction. Our results show substantially fewer high-abundance proteins, consistent with less contamination. As expected with the outstanding sensitivity of current MS instruments, we were able to detect low-abundance proteins with the current proteome.

### Functions of bona fide LD proteins

**Fig. 4.**
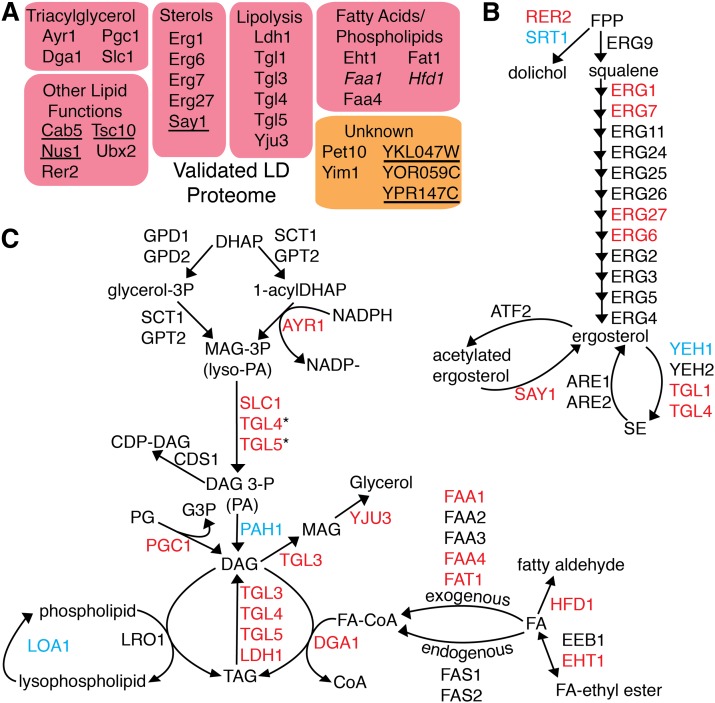
Functional annotation of identified LD localized proteins. A: All validated LD proteins with known functions are involved in LD metabolism. Newly verified proteins are in italics. Newly identified proteins are underlined. B: Proteins that copurify with LDs include many enzymes in sterol metabolism. Two are newly identified (Rer2 and Say1). C: Proteins that copurify with LDs include most enzymes in TG metabolism. *Activity shown in vitro, presumed to be minor function in vivo. Proteins marked in red were identified and microscopically verified in the current work. Proteins marked in blue were not identified in our proteome but have been microscopically verified in other works.

We annotated the functions of the 30 bona fide LD proteins based on available data in the literature (**Fig. 4A**). Interestingly, all proteins with known functions (83%) are involved in lipid metabolism. These included many proteins involved in ergosterol metabolism (Fig. 4B) and FA esterification and TG metabolism (Fig. 4C). The only de novo TG synthetic enzyme that we failed to detect in our LD proteome is Pah1, although it has previously been localized to the LD (36). Four of our six newly identified LD proteins increase the variety of lipid metabolic functions ascribable to the LD: Cab5 is involved in CoA synthesis (37), an important cofactor for many lipid synthesis reactions; Tsc10 catalyzes the second step in the synthesis of phytosphingosine, a long-chain base for sphingolipid production (38); Rer2 and Say1 function in dolichol and sterol metabolism, respectively, and are discussed below. Five proteins (Pet10, Yim1, YOR059C, and the newly identified YKL047W and YPR147C) do not have clear functions in yeast (see Table 1), but are likely to be involved in lipid metabolism as well. The mammalian ortholog of YPR147C was recently reported to have cholesterol esterase activity (39).

Of the six newly identified LD proteins, we chose to focus our further investigations on Rer2 and Say1 because they have known enzymatic functions in sterol metabolism, and their connections to LDs have not been well explored.

### Rer2 is active in the LD fraction

Rer2 is a *cis*-IPTase involved in dolichol synthesis (40). It is a 286-amino acid protein with a predicted globular structure. *cis*-IPTases condense successive IPPs with FPP to create polyprenols, such as dolichol, the lipid anchor for sugars used in N-linked glycosylation. Because many enzymes in N-glycan biosynthesis were recently identified at the *Drosophila* LD (25), we sought to better understand the LD localization of Rer2.

**Fig. 5.**
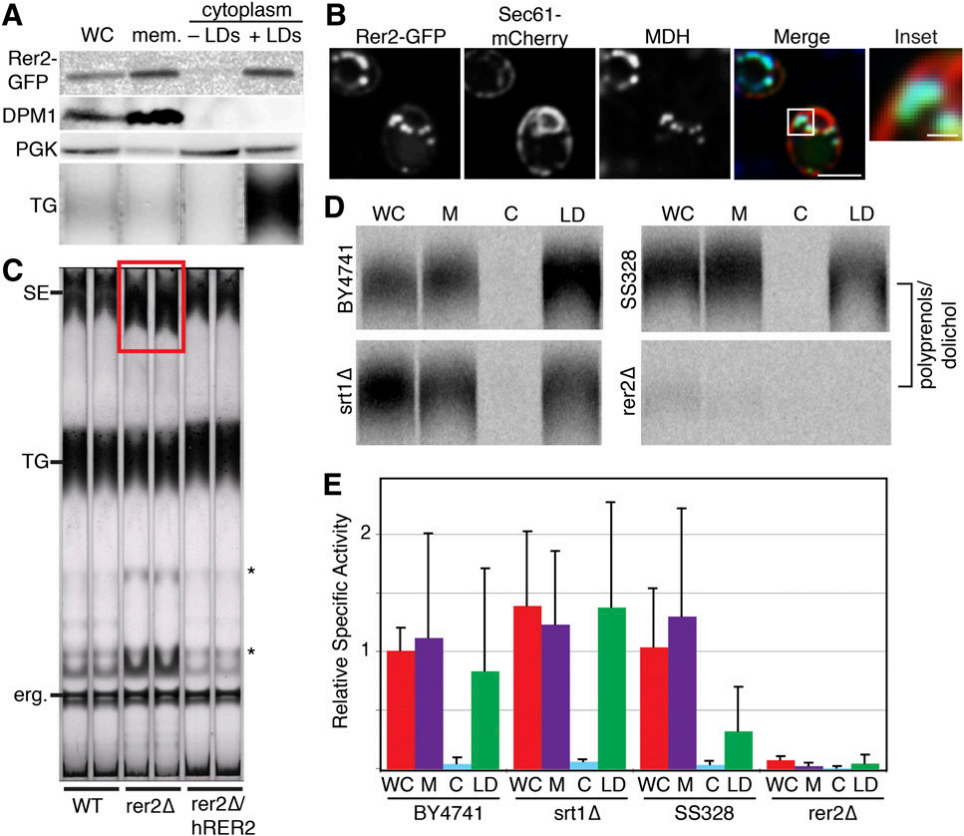
Rer2 is present and active at the LD. A: Rer2-GFP is present at the LD and in membranes by Western blot. Rer2-GFP-labeled cells were centrifuged at 100,000 *g*. LDs were collected by slicing centrifuge tubes. The upper fraction (containing LDs) was rinsed and the rinsed upper fraction, lower fraction (containing cytoplasm), and pellet (containing membranes) were probed with anti-GFP antibody (for Rer2), anti-phosphoglycerate kinase (PGK) antibody (for cytoplasm), and anti-dolichol phosphate mannose synthase (DPM1) antibody (for ER). The lipids were extracted and separated by TLC. TG was identified by comigration with a standard. B: Rer2-GFP is present at the LD and ER by spinning disk confocal microscopy. Rer2-GFP shows a reticular and punctate pattern that colocalizes with ER marker Sec61-mCherry and LD stain MDH. C: The *rer2Δ*s have increased levels of SEs (red box) as determined by TLC. Expression of DHDDS (human homolog of Rer2) rescues the SE accumulation. Unidentified lipids are marked with a *. D: Rer2 is active at the LD. Cell extracts, as in (A), were given FPP and ^14^C-IPP, which were incorporated into polyprenols by *cis*-IPTases in both the membrane and LD fractions. *cis*-IPTase activity is nearly WT in an *srt1Δ* strain and nearly missing in an *rer2Δ* strain, suggesting that Rer2 is the major *cis*-IPTase in both microsomes and LDs. BY4741 is the WT strain for the *srt1Δ* strain and SS328 is the WT strain for the *rer2Δ* strain. E: Quantification of (D), normalized to WT whole cell activity. Data are the mean ± standard deviation of n = 4–8 samples. There are no statistical differences between individual fractions in *srt1Δ* and BY4741. Whole cell and membrane *cis*-IPTase activity is significantly reduced (*P* < 0.05) in *rer2Δ* when compared with SS328. WC, whole cell; M or mem., membrane; C, cytoplasm.

There are two known *cis*-IPTases in yeast, Rer2 and Srt1. Another protein, Nus1, has significant homology to Rer2 and Srt1, but does not have *cis*-IPTase activity (41). Both Srt1 (42) and Nus1 (22, 37, 43) have been localized previously to the LD. We found that Rer2 also localizes to the LD, as we detected Rer2-GFP in both membrane and LD fractions (**Fig. 5A**) and saw colocalization of Rer2-GFP with ER marker Sec61-mCherry and MDH (Fig. 5B). The deletion of Rer2 affects neutral lipids, as *rer2*Δ cells showed a specific increase (>2-fold) in SE levels (Fig. 5C) that was rescued by expression of the human Rer2 homolog, dehydrodolichyl diphosphate synthase (DHDDS).

To assess whether Rer2 is active at LDs, we examined *cis*-IPTase activity in cell extracts by incubating cells with FPP and ^14^C-IPP and measuring incorporation into polyprenols by TLC. We found that WT cells had *cis*-IPTase activity in both membrane and LD fractions (Fig. 5D). The *srt1*Δ cells had similar *cis*-IPTase activities, while *rer2Δ* cells had very little *cis*-IPTase activity in either membrane or LD fractions, indicating that Rer2 is the major source of polyprenol synthesis at the ER and the LD in stationary phase yeast.

### Say1 is active in the LD fraction

**Fig. 6.**
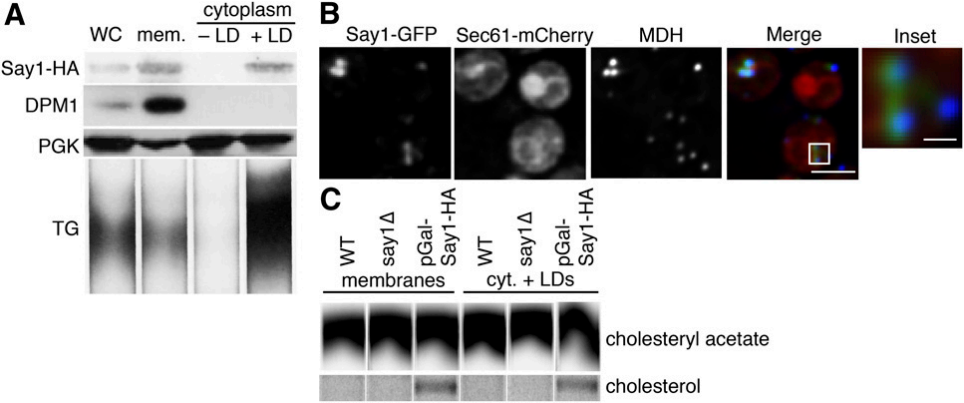
Say1 is present and active at the LD. A: Say1-GFP is present at the LD and in membranes by Western blot. pGal-Say1-HA cells were fractionated as in Fig. 5 and probed with anti-HA antibody (for Say1), anti-PGKantibody (for cytoplasm), and anti-DPM1 antibody (for ER). Lipids were extracted and separated by TLC. TG was identified by comigration with a standard. B: Say1-GFP is present at the LD and ER by spinning disk confocal microscopy. Say1-GFP shows a reticular and punctate pattern that colocalizes with ER marker Sec61-mCherry and LD stain MDH. C: Say1 is active at the LD. Cell extracts were given ^14^C-CA, which was deacetylated into free cholesterol in both membrane and LD fractions by cells grown in galactose and overexpressing Say1-HA under a GAL1 promoter. CA deacetylase activity was below the limit of detection in WT or *say1Δ* cells. WC, whole cell; M or mem., membrane; C or cyt., cytoplasm.

In yeast, sterol metabolism is intricately linked between LDs and the ER. We therefore further investigated Say1, a sterol metabolic enzyme that we newly identified as a LD protein. While Say1 has been previously localized to the ER, we detected Say1-GFP in both the ER and LD fractions (**Fig. 6A**). Additionally, Say1-GFP colocalized with ER marker Sec61-mCherry and MDH, showing that it has both ER and LD localization (Fig. 6B). The *say1*Δ yeast do not have grossly altered levels of SE or TG (data not shown).

Yeast cells apparently have a cycle of sterol acetylation and deacetylation wherein Atf2 promiscuously acetylates sterols so they can be secreted in a detoxification mechanism while Say1 specifically deacetylates acetylated sterols that should be retained within the cell, like ergosterol and its synthetic intermediates (44). To measure Say1 activity, we fractionated cells, added radiolabeled CA, and monitored the appearance of free cholesterol. We detected sterol deacetylase activity in both ER and LD fractions (Fig. 6C, D) when we overexpressed Say1-HA under control of a *GAL1*-promoter. Thus, it appears that Say1-mediated sterol deacetylation is present at the LD. We did not detect sterol deacetylase activity in either WT (cells not overexpressing Say1-HA) or *say1Δ* cells, consistent with a previous report that was only able to detect deacetylation activity on an artificial substrate when Say1 was overexpressed (44).

## DISCUSSION

### A yeast LD proteome generated by PCP

LD proteomes are challenging to generate because of the hydrophobic nature of the LD and its close apposition to other organelles. Although LD proteomes have consistently identified a core set of important LD proteins, they are plagued with false positives from copurifying contaminants. Previous MS-based proteomic examinations of yeast LD proteins yielded lists of candidates ranging in number from 76 (22) to 440 (45). PCP provides a method to determine a yeast LD proteome that is both highly sensitive and specific (25). In the current study, we applied PCP to the analysis of yeast LDs and identified 35 proteins that reproducibly and stringently copurify in the LD fraction. Of the 35 proteins identified, 30 of these proteins localize to the LD by microscopy, in our studies and others (Fig. 2, Table 1), identifying them as bona fide LD proteins. Importantly, we identify six proteins (Cab5, Rer2, Say1, Tsc10, YKL047W, and YPR147C) that had not previously been localized to the LD. Further, we show that two of these proteins, Say1 and Rer2, are lipid metabolic enzymes that are active in LD fractions. One of the proteins that we newly identified, Cab5, is involved in CoA biosynthesis with Cab2, Cab3, and Cab4 (37). Although none of Cab2–4 were identified as LD proteins in our proteome, by fluorescence microscopy, we were additionally able to detect Cab4-GFP at the LD (data not shown). Using fluorescence microscopy, we also verified stationary-phase LD localization of two proteins previously identified in multiple LD proteomes and identified in our proteome, but never visually shown to be at the LD (Faa1 and Hfd1).

Our results differ somewhat from previous reports of yeast LD proteomes (see Fig. 3). The first reported LD proteome identified only 19 proteins (34), in part due to technological limitations of MS at the time, but was likely highly specific and most closely overlaps with the current results. Two subsequent proteomes benefited from enhanced MS sensitivity, but had limited confirmation and likely included a number of contaminants, particularly of highly abundant proteins. The lack of overlap between the proteomes may also reflect differences in culture conditions [Binns et al. (11), minimal media with oleate; Grillitsch et al. (22), rich media with and without oleate; Athendstaedt et al. (34), rich media without oleate] or differences in purification methods. The twelve proteins identified in all proteomes (Ayr1, Eht1, Erg1, Erg6, Erg7, Faa1, Faa4, Fat1, Pet10, Slc1, Tgl1, and Tgl3) are likely to be constitutive LD proteins, because they were identified regardless of specific growth conditions.

Although our PCP identified 35 proteins that specifically copurify with the LD, we were unable to verify LD localization by fluorescence microscopy for five proteins (Fig. 2, Table 1). For some of these proteins, GFP-tagging might interfere with their targeting to the LD. For Gtt1, microscopy revealed that clusters of Gtt1-GFP localize near some LDs, offering an explanation for why Gtt1 biochemically purifies with LDs. Taz1, a cardiolipin remodeling enzyme found primarily in mitochondrial membranes (46), might also copurify with LDs due to organelle associations.

Our proteome failed to identify some bona fide LD proteins [e.g., Atf1 (47), Loa1 (48), Pah1 (36), Pdr16 (49), or Srt1 (42)]. That we did not identify some proteins may reflect the dynamic nature of protein localization to the LD [we only examined stationary phase cells grown in minimal media and nutrient carbon source affects protein targeting to the LD (22)], limitations of organelle fractionation, or our stringent filtration criteria. Our stringent criteria potentially filtered out several proteins that were present in the LD and other non-ER cellular fractions, as it did for Pdr16, which localizes to the LD and cell periphery (49). Atf1 and Srt1 were not detected in the LD fraction, whereas Loa1 was only detected in a single replicate. Pah1 was found in the LD fraction but not included because it lacked sufficient H/Ls in multiple fractions, as our criteria demanded.

Our PCP analysis of the yeast LD proteome revealed some notable differences from a PCP analysis of LDs in *Drosophila* S2 cells (25). Although both PCP studies found that the majority of LD proteins function in lipid metabolism, we found considerably more LD proteins (>100) in *Drosophila* cells. The additional proteins are involved in ER organization, protein degradation, and N-glycan biosynthesis, suggesting additional functions and complexity for LDs in fly versus yeast cells. Other differences might relate to the different relationships between the ER and LDs in these cell types. In yeast, LDs are often more directly connected or exist as a subdomain of the ER (17), whereas in S2 cells there are distinct LD populations, those that are connected and not connected to the ER (9). The closer association of LDs and ER in yeast may explain why this study required additional filtration criteria to yield a LD-specific list of proteins.

A specific list of yeast LD proteins presents the opportunity to determine how these proteins target to the LD. For example, of the 12 proteins found in all reported proteomes, six have predicted transmembrane domains (Erg1, Erg7, Fat1, Slc1, Tgl1, and Tgl3), with the topology of Tgl1 experimentally verified (50). It is unclear how a protein with a transmembrane domain can localize to a membrane monolayer at LD surfaces. In yeast, perhaps many of these transmembrane proteins are in an ER microdomain that is closely associated with the LD and indistinguishable by biochemical fractionation or light microscopy. LDs appear to be a subdomain of the ER in yeast, and LD-ER bridges have been found in yeast and a number of other organisms (9, 17, 51, 52).

Most of the newly identified yeast LD proteins (Cab5, Rer2, Say1, Tsc10, and YPR147C) have human orthologs; and thus, the localization to LDs might also be important for functions of these proteins in humans. The functional homolog of Rer2, DHDDS (or hCIT), has been linked to retinitis pigmentosa (53). Human CoA synthase is a bifunctional enzyme (phosphopantetheine adenylyltransferase and dephospho-CoA kinase activities) that catalyzes the last step of CoA synthesis, like Cab5 (54). While sterol acetylation is a yeast-specific process, Say1 is orthologous to arylacetamide deacetylase (44), an enzyme putatively involved in TG hydrolysis (55). Tsc10 is functionally homologous to 3-ketodihydrosphingosine reductase (FVT1), known to be active at the cytosolic face of the ER (56) and implicated in tumor processes (57). YPR147C is a highly conserved protein with a GXSXG lipase motif that has been shown to affect lipid storage in *Drosophila* (58) and cholesterol ester storage in macrophages (39).

### Identification of Rer2 and Say1 as LD-localized enzymes in yeast

Our findings show that the *cis*-IPTase Rer2 localizes in part to LDs. *cis*-IPTase activity is an essential step in dolichol biosynthesis and in yeast is catalyzed by Rer2 and Srt1, with Nus1 as a potential cofactor (41). Both Srt1 and Nus1 were previously reported at the LD (11, 22, 42, 43). We found that the *cis*-IPTase Rer2 localizes to the ER and LDs both biochemically and by fluorescence microscopy, and has similar specific activities in each compartment. The major role of ER-localized Rer2 is to generate dolichol for glycosylation, and yeast lacking LDs [lacking all four enzymes of neutral lipid synthesis (59)] still make dolichol (not shown). Therefore, LD-localized Rer2 may be more important for synthesizing dolichol destined for storage pools or other cellular functions.

It is unclear how Rer2 localizes to LDs. Rer2 does not have predicted transmembrane sequences, although it behaves as an integral membrane protein (40). Thus, there are no theoretical topological problems for its localization to the LD. In our experimental conditions, Rer2 appears to be the major cellular *cis*-IPTase. A previous report showed that Srt1 was most highly expressed in stationary phase and Srt1-dependent activity was detected in an *rer2Δ* background when Srt1 was overexpressed and only in stationary phase (42). However, we found little *cis*-IPTase activity in the *rer2Δ* in stationary phase, leaving questions about the cellular role of Srt1.

Yeast cells with Rer2 deletion exhibited an increase in SEs. While stressed or slow-growing yeast cells often accumulate LDs, the accompanying neutral lipid accumulation is usually both SE and TG. We suspect that *rer2*Δ cells likely have a SE-specific accumulation because FPP, a Rer2 substrate, may be channeled into sterol synthesis and SEs. Rechanneling of FPP is consistent with the finding of several unidentified radiolabeled lipid species synthesized by *rer2Δ* cells in cisIPTase activity assays (data not shown).

We also found that an enzyme involved in a sterol detoxification system, Say1, targets to the LD. A current model suggests that Say1 works in concert with Atf2. Atf2 promiscuously acetylates exogenous and endogenous sterol molecules, which are then secreted unless they are recognized and deacetylated by Say1 (44). The topology of ER-localized Say1 showed that the enzyme has a single transmembrane domain (44). Such a topology should not exist at the LD monolayer because it would put a hydrophilic protein domain in the hydrophobic core of the LD. This suggests that LD-localized Say1 is a component of ER that is tightly associated with LDs, a possibility that is consistent with the apparent connections of ER and LDs in yeast (17).

Rer2 and Say1 join a list of lipid metabolic enzymes that localize to LDs. While both Say1 and Rer2 are present and active at the LD and ER, similar to Ayr1 (60), Gpt2 (61), and Slc1 (62), not all proteins are active in all subcellular populations. For example, Dga1 (63), Erg6 (64), Erg7 (65), Erg27 (66), and Yju3 (67) are predominantly active at the LD, which may reflect that these proteins have higher LD:ER localization ratios. In contrast, Erg1 is strongly present in both the ER and LD but only active at the LD (68). The significance of some enzymes differing in activity in the ER versus LDs is unclear.

## Supplementary Material

Supplemental Data
