# Supplementary material for: High confidence proteomic analysis of yeast LDs identifies additional droplet proteins and reveals connections to dolichol synthesis and sterol acetylation
Source: J Lipid Res. 2014 Jul;55(7):1465–77. doi: 10.1194/jlr.M050229 (PMC4076087; doi:10.1194/jlr.M050229)
Supplement: Supplemental Data [file supp_55_7_1465__index.html]

High-Confidence Proteomic Analysis of Yeast Lipid Droplets Identifies Additional Droplet Proteins and Reveals Connections to Dolichol Synthesis and Sterol Acetylation — High confidence proteomic analysis of yeast LDs identifies additional droplet proteins and reveals connections to dolichol synthesis and sterol acetylation — Supplemental Data 

# High confidence proteomic analysis of yeast LDs identifies additional droplet proteins and reveals connections to dolichol synthesis and sterol acetylation

## Supplemental Data

**Files in this Data Supplement:**

- Supplementary Table 1 - Proteome dataset
- Supplementary Table 2 - Proteins with membership value of &#x26;gt;.1 in LD fraction
